# A case of hepatocellular carcinoma with skin injury of the upper abdominal wall after transcatheter arterial chemoembolization: a case report

**DOI:** 10.4076/1757-1626-2-7197

**Published:** 2009-09-01

**Authors:** Hiromitsu Kanzaki, Kazuhiro Nouso, Koji Miyahara, Naoko Kajikawa, Sayo Kobayashi, Ichiro Sakakihara, Shota Iwadow, Shuji Uematsu, Ryoichi Okamoto, Kunihiro Shiraga, Motowo Mizuno, Yasuyuki Araki

**Affiliations:** Department of Internal Medicine, Hiroshima City Hospital7-33, Moto-machi, Naka-ku, Hiroshima-city, Hiroshima, 730-8518Japan

## Abstract

**Introduction:**

Transcatheter arterial chemoembolization has been widely used to treat advanced hepatocellular carcinoma that cannot be treated by local ablation therapies or surgical resection. The effectiveness of transcatheter arterial chemoembolization in prolonging survival has been well established, and approximately one third of newly discovered hepatocellular carcinoma patients were repeatedly treated by transcatheter arterial chemoembolization in Japan. Various kinds of complications have been reported, and many of which are general complications such as hepatic coma, jaundice, fever-up, ascites, and bile duct injury.

The hepatic falciform artery is found frequently during postmortem anatomic dissection and the incidence of hepatic falciform artery is reported to be over 60%. Hepatic falciform artery is known to be the responsible artery for supraumbilical skin rash development after arterial chemo infusion therapy; however, skin complications after transcatheter arterial chemoembolization are rare.

**Case presentation:**

A 70-year-old female with chronic hepatitis C infection was diagnosed as having hepatocellular carcinoma (S4, 20 mm in diameter). Transcatheter arterial chemoembolization was performed via the left hepatic artery, which was a feeding artery of the hepatocellular carcinoma. Two days after that, supraumbilical skin rash with local tenderness and redness appeared. Retrospective analysis revealed that occlusion of the hepatic falciform artery branching from the left hepatic artery with micromaterials caused the skin lesion.

**Conclusion:**

We should keep in mind that anticancer drugs or embolic materials can flow into the HFA and may cause abdominal wall injury after transcatheter arterial chemoembolization.

## Case presentation

A 70-year-old Japanese female (Asian) had suffered from chronic hepatitis C since 1992. In March 2006, hepatocellular carcinoma (HCC) (20 mm in diameter) was found in liver segment 4, and she was admitted to our hospital. She had a history of tuberculosis of the lymph nodes in 1992.

Laboratory findings on admission were as follows: white blood cell count 1.77 × 1000/mm^3^, red blood cell count 339 × 104/mm^3^, hemoglobin 11.3 g/dL, hematocrit 31.9%, platelet count 3.1 × 10000/mm^3^, total protein 7.3 g/dL, albumin 3.5 g/dL, total bilirubin 1.7 mg/dL, direct bilirubin 0.7 mg/dL, aspartate aminotransferase 68 IU/L, alanine aminotransferase 35 IU/L, alkaline phosphatase 402 IU/L, γ-glutamyltranspeptidase 13 IU/L, cholinesterase 108 IU/L, total cholesterol 137 mg/dL, blood urea nitrogen 16 mg/dL, Creatinine 0.7 mg/dL, Na 142.5 mEq/L, K 3.0 mEq/L, Cl 110.2 mEq/L, Ca 8.8 mg/dL, prothrombin time 63.8%, ICG-R 33.8%, ICG-K 0.072, des-gamma-carboxy prothrombin 325 mAU/mL, and alpha-fetoprotein 6.8 ng/mL. The patient was positive for HCV antibody but negative for hepatitis B virus surface antigen.

**Figure 1.**
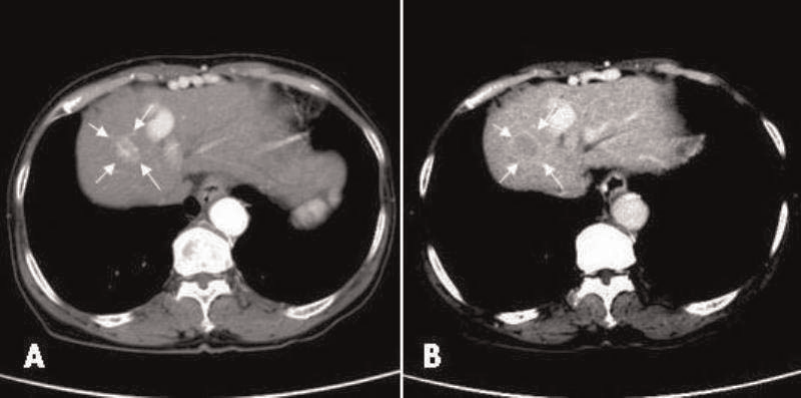
Axial computed tomography image with contrast enhancement before angiography. Classical HCC (20 mm) was observed in segment 4 (arrows). Collateral veins were seen along the abdominal wall (arrow heads). A; Early phase. B; Delayed phase.

Enhancement in the early phase and perfusion defect in the delayed phase were observed in segment 4 (20 mm in diameter) of the liver by computed tomography, and collateral veins were also noted (Figure 1). The tumor findings by abdominal ultrasonography were typical of HCC, which was a hyperechoic space occupied by a lesion with hallow. Hepatic arteriography showed tumor staining in the delayed phase, and the feeder of the lesion was A4 (artery 4). Lipiodol (2 ml) mixed with epirubicin (12 mg) followed by gelatin sponge was injected via the left hepatic artery, and A4 was completely occluded.

**Figure 2.**
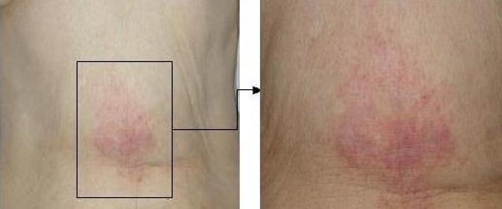
Skin lesion at 3 days after TACE. The patient complained of epigastric pain soon after TACE, and the pain persisted for 5 days. Skin redness appeared on the upper abdominal wall on day 2.

**Figure 3.**
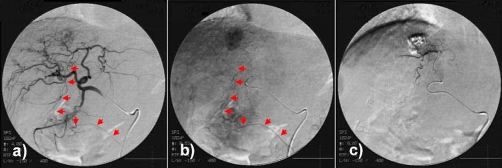
**(A)** Arterial phase of the left hepatic arteriogram showing a dilated falciform artery (arrows) arising from the left hepatic artery. **(B)** In the delayed phase, the flow persisted, and HCC staining was clearly detected in segment 4. **(C)** The HFA shadow completely disappeared after TACE.

Soon after Transcatheter arterial chemoembolization (TACE), she complained of epigastric pain, and a skin rash appeared on the upper abdominal wall on day 2 (Figure 2). Angiography was retrospectively re-evaluated. The HFA branching from the left hepatic artery was faintly observed in the early arterial phase, and the flow persisted until the delayed phase (Figure 3). After TACE, hepatic falciform artery (HFA) had completely disappeared. The area of the skin lesion was consistent with the perfusion area of the HFA, so that inflow of the anticancer drug to the HFA and its subsequent occlusion were presumed to be the cause of the skin rash.

**Figure 4.**
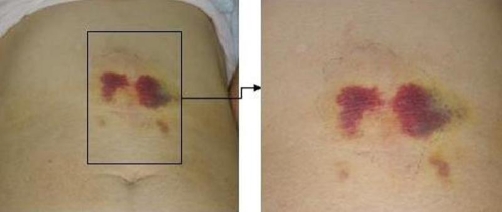
After the skin rash and pain were resolved, purple spot and hard subcutaneous nodule was emerged.

We injected steroid in the site on day 3 and 7, and prescribed tranilast (anti-allergic agent). At 5 days after TACE, the skin rash and pain resolved, and purple spots and hard subcutaneous nodules emerged on day 7 (Figure 4). Ten months later, the skin rash had completely healed.

## Discussion

The HFA arises as a small terminal branch of the left or middle hepatic artery, runs through the hepatic falciform ligament, distributes itself around the umbilicus, and communicates with branches of the superior and inferior epigastric arteries [7-13]. Michels et al. reported that the HFA is found frequently during postmortem anatomic dissections, and that the incidence of HFA was 39 of 58 patients (67.2%) [13]. There are some reports on skin complications in angiography caused by the inflow of anti-cancer drugs or occlusion materials to HFA [7-12]. The complication is rare and does not necessarily occur in all cases with HFA. However, once skin injury has occurred, it is sometimes severe enough to require debridement. Therefore, we should always pay attention to the artery during transcatheter arterial treatments.

There are two major factors concerning skin injury due to transcatheter therapies. One is the size of the occlusion material: small particles like Lipiodol sometimes embolize the perfusion area completely [11-12]. The other is the duration of chemotherapeutic agents: continuous arterial injection of anti-cancer drugs is known to induce skin injury [10]. We used Lipiodol mixed with epirubicin, and the dose was the same as we routinely use, so that the reason for the skin lesion seems different from the two reported factors. It is known that portal hypertension and a decrease in portal vein flow cause a compensatory increase of hepatic arterial flow and the development of portal collateral vessels [7]. Our case showed severe collateral vein accompanied by portal hypertension. Therefore, it is possible that HFA (collateral vein) flow increased, and the volume of chemical micromaterials flowing into the HFA was enhanced, resulting in skin injury.

While high incidence of HFA (67.2%) was reported on postmortem anatomic dissections, the reported incidences of HFA on angiography were low (ranging from 2% to 24.5%) [7-9]. One of the reasons for these discrepancies seems to be the velocity of arterial flow. The flow was often too slow to be regularly detected during the early arterial phase on angiography [9]. The similarity of the HFA route with those of other arteries might be another reason. The HFA runs close to the hepatic artery, cystic artery, and gastroduodenal artery, so that it is easy to misinterpret the HFA.

There are controversies with regard to the relationship between the HFA and the hepatic disease state. Kim et al. reported that patients with an HFA had significantly higher serum bilirubin levels, lower albumin levels, and longer prothrombin times than patients without one. Chung et al. reported that portal vein collateral vessels were more frequently observed in patients with HFA presence than absence [6]. Conversely, Gibo et al. reported no significant difference in HFA incidence between a chronic liver disease group and normal liver group [8]. Although the mechanism of the skin injury in our case can be explained by Chung’s report, further study is needed to reach a conclusion regarding these discrepancies.

For the prevention of skin rash, it is reasonable to perform TACE by placing the tip of the microcatheter beyond the origin of the HFA, or embolization of the HFA with microcoils or gelatin sponge prior to TACE. Because the HFA often communicates with branches of inferior and superior epigastric arteries, the skin lesion does not occur when embolizing the HFA with micro coils or simple gelatin sponge particles [8-12]. Hence, it is reasonable to consider embolizing the HFA with micro coils to prevent such a complication, especially in cases with severe portal hypertension or undergoing long-term chemotherapy with a reserved microcatheter, in which the drug volume needs to be increased.

In conclusion, we should keep in mind that anticancer drugs or embolic materials can flow into the HFA and may cause abdominal wall injury after TACE.

## Abbreviations

HCC: hepatocellular carcinoma
HFA: hepatic falciform artery
TACE: transcatheter arterial chemoembolization
